# Editorial: Non invasive Stimulation Techniques: “Modulating Cognition”

**DOI:** 10.3389/fpsyg.2019.00922

**Published:** 2019-04-24

**Authors:** Olga Lucía Gamboa

**Affiliations:** ^1^Department of Neurology and Brain Imaging Center, Goethe University, Frankfurt am Main, Germany; ^2^Department of Psychology and Neuroscience, Duke University, Durham, NC, United States

**Keywords:** Non-invasive brain stimulation, TMS, tDCS, sham, cognition

Over the last years non-invasive brain stimulation techniques (NIBS) have become the ultimate tool to gain major insights about the mechanisms responsible for sensory, motor, and cognitive functions. A big issue surrounding transcranial magnetic stimulation (TMS) and transcranial electric stimulation (TES) methods is the disagreement about the aftereffects reported by studies using similar (if not the same) stimulation protocols (Robertson et al., 2003; Horvath et al., 2014). The purpose of this research topic was to collect information regarding different stimulation procedures to assess their capacity to modulate cognition including also, appropriate control and sham conditions. The first part of this report will cover contributions related to TES which were limited to transcranial direct current stimulation methods (tDCS). This will be followed by studies dedicated to real TMS and sham methodology.

Berryhill et al. scrutinized the characteristics of studies using tDCS to modulate cognitive function aiming to provide some guidelines for this type of studies. The factors proposed by the authors to ensure high quality and reproducibility of cognitive studies using tDCS include: (1) Recruitment of a large and homogeneous population to amplify the typically small effects size in tDCS-cognitive studies and to control for different stimulation effects in the population. (2) Presentation of challenging and engaging cognitive tasks to profit from the relationship between tDCS effects and task difficulty. (3) To control for the participant's level of motivation, since poor motivation may obscure tDCS after effects. Additionally, Berryhill and colleagues allude to the file drawer problem in the tDCS literature as an obstacle in establishing a solid frame to understand the brain mechanisms underlying tDCS interventions.

Another factor to consider was proposed by Moreau et al.. According to their findings combining tDCS with physical exercise has the potential to facilitate learning processes as long as the brain region under stimulation has not reached an optimized level of activation. They argued that tDCS after–effects may be too small to further enhance high performance.

In a double blind study Antal et al. investigated the modulatory effects of anodal and cathodal tDCS applied over the visual cortex during a reading task. Unexpectedly, enhanced excitability induced by cathodal stimulation was observed. Modulatory neural processes resembling metaplasticity caused by the stimulation alone or together with the ongoing task learning were proposed as the possible reasons behind this finding. Based on their general results, the authors suggested that the lack of correct acknowledgement of the participant's cognitive state during the stimulation session could be an important element adding up to the conflicting outcomes reported from stimulation studies. To minimize discrepancies observed in tDCS studies and optimize the stimulation method, the authors recommend standardizing the conditions during the experimental session, particularly defining the range of cognitive activities unrelated to the task that participants are allowed to perform and to methodically control for environmental parameters.

Although the previous contributions have focused on the area of TES it would be fair to say that the findings and suggestions described above are well applicable to TMS methodology. However, the following contributions elaborate their findings in the TMS domain.

The work of Salatino et al. proposed an inexpensive and straightforward method to determine the optimal stimulation site of the posterior parietal cortex (PPC), so that visuospatial perception in healthy participants can be modulated through TMS during a line length estimation task. They observed a high variability at the individual level, associated to the stimulation site where TMS showed an effect. The authors suggested that these differences may be caused by anatomical and functional differences between participants. This point could be one of the reasons why reproducibility of TMS aftereffects has been challenging. Unfortunately, dealing with this issue is not always an easy task, perhaps due to insufficient budget or resources.

In a mini-review Schuwerk et al. presented the undeniable value of rTMS procedures in advancing the knowledge of the neurocognitive mechanisms behind mentalizing with a focus on the impairment observed in disorders such as autism and major depression. Interestingly, in the examined literature, the authors found that, regardless of the stimulation method used (single pulse TMS, online/offline rTMS or tDCS), most of the studies reported relevant aftereffects when compared to either sham or control conditions. However, they also identified some limitations, such as poor description of the employed methods and the often undisclosed TMS effect size. Lastly, they pointed out the relevance of developing cognitive tasks sensitive enough to detect the effects of TMS on behavior.

Finally, Duecker and Sack elegantly presented their views on the different methodological issues surrounding sham TMS. In their opinion the best option to lessen detrimental placebo effects is to adopt effective blinding methods for TMS, so that participants are not able to distinguish between real and sham conditions and to use electrical stimulation combined with a sham TMS coil to best mimic the peripheral nerve stimulation and the auditory and somato-sensory effects caused by active TMS. The authors also stressed the value of using additional control strategies to evaluate brain specificity during TMS interventions. Thus, in an ideal scenario a typical TMS experiment should include active TMS, an active control condition and a sham condition for each brain site actively stimulated. Furthermore, Duecker and Sack encourage data sharing of the results regarding blinding success as a means to improve the quality of TMS methods.

Despite the recent advances in NIBS research using concurrent neuroimaging methods, the mechanisms behind this technology are still unclear. This is likely in part due to the lack of reproducibility of reported results. This, added to the rapid growth of NIBS being used as a therapeutic tool reinforces the need to follow more rigorous stimulation procedures following guidelines as the ones presented in this topic. Accounting for participant's variability, controlling environmental, cognitive, and methodological factors (sham–control strategies) and disclosing detailed experimental reports are important steps to optimize stimulation methods and to increase the chances of obtaining reliable and reproducible results.

## Author Contributions

The author confirms being the sole contributor of this work and approved it for publication.

### Conflict of Interest Statement

The author declares that the research was conducted in the absence of any commercial or financial relationships that could be construed as a potential conflict of interest.
